# Clinical Lecture in the Ophthalmic Department of the Cook County Hospital

**Published:** 1874-11-15

**Authors:** F. C. Hotz


### Clinical Reports.

### CLINICAL LECTURE IN THE OPHTHALMIC DEPARTMENT OF THE COOK CO. HOSPITAL.

By F. C. Hotz, M.D.

*Reported by F. C. Winslow, M. D., Assistant Physician.*

GENTLEMEN: I invite your attention to-day to a case of Convergent Strabismus, or squint, in which condition the visual axis of the affected eye is directed to a point much nearer than the object at which the person looks. We are indebted to Professor Donders for our knowledge of the fact that convergent strabismus is usually the result of hypermetropia.

The affection is usually developed in children between three and five, and is contemporaneous with their first efforts at observation. It should receive early and prompt attention, because if allowed to exist for many years the sight of the affected eye is injured and ultimately lost. This is owing to the fact that the image of the object is, by the distortion of the position of the eye, thrown upon the periphery of the retina, where the impression it makes is so feeble that the mind of the child is unable to take cognizance of it, and it is soon entirely disregarded.

The relief for this condition is the division of the tendon of the internal rectus, and it is to this operation that the patient before us proposes to submit.

She is a lady aged twenty. Has had convergent strabismus sixteen years. She says she has been operated upon once, but the result was not particularly favorable. We administer ether in this case on account of the timidity of the patient, although in persons of moderate firmness it is not necessary. Operating while the patient is anæsthetized is less advantageous from the fact that if it becomes necessary to correct the position of the other eye, as it often is in cases of long standing, it is impossible to do this until the patient has fully regained consciousness. This delays the second operation unnecessarily. The lids are separated with the speculum, and a fold of the conjunctiva over the external rectus seized in a pair of fine-toothed forceps. These are passed to an assistant who thus controls the movements of the eye. The organ is then rotated well outward and the conjunctiva divided vertically with the scissors over the insertion of the internal rectus. A blunt hook is now passed through the wound and around the tendon of the external rectus, which is dissected loose from its attachments by means of the scissors. The blunt hook is then passed back and forth under the conjunctiva up to the margin of the cornea, in order that we may be sure that no lateral attachments remain undivided. No dressing is necessary, but the patient is directed to return in two days. At this time it was found necessary to treat the other eye in the same manner, to correct the deviation of this organ also.

The second operation was performed without an anæsthetic. This case, you observe, is entirely relieved of the deformity, the visual axis of both eyes being now directed to the object.

The tendency of the eye to rotate outwards after division of the tendon of the internal rectus must make us cautious in the operation, lest we weaken the muscle to such a degree that it will be unable to control the movements of the eye. This is a most disastrous result, for, while a slight convergence is often disregarded by both patient and observer, the least deviation in the way of divergence is very noticeable. Therefore, if after both operations a slight convergence still remains we are not justified in repeating the division, as it often occurs that in extreme cases we must be satisfied with a result which may not be exactly perfect.

---

